# Real‐world treatment pattern and prognostic factors of stage IV lung squamous cell carcinoma patients

**DOI:** 10.1002/kjm2.12599

**Published:** 2022-10-10

**Authors:** En‐Chi Hsu, Kuan‐Li Wu, Ying‐Ming Tsai, Mei‐Hsuan Lee, Ming‐Ju Tsai, Chia‐Yu Kuo, Yu‐Chuan Liu, Fu‐Wen Liang, Chih‐Jen Yang, Jen‐Yu Hung

**Affiliations:** ^1^ Division of Pulmonary and Critical Care Medicine, Department of Internal Medicine Kaohsiung Medical University Hospital, Kaohsiung Medical University Kaohsiung Taiwan; ^2^ Graduate Institute of Clinical Medicine, College of Medicine Kaohsiung Medical University Kaohsiung Taiwan; ^3^ School of Medicine, College of Medicine Kaohsiung Medical University Kaohsiung Taiwan; ^4^ Department of Internal Medicine Kaohsiung Municipal Siaogang Hospital, Kaohsiung Medical University Kaohsiung Taiwan; ^5^ Clinical Trial Center, Kaohsiung Medical University Hospital Kaohsiung Medical University Kaohsiung Taiwan; ^6^ Department of Public Health Kaohsiung Medical University Kaohsiung Taiwan; ^7^ Department of Internal Medicine, Kaohsiung Municipal Ta‐Tung Hospital Kaohsiung Medical University Kaohsiung Taiwan

**Keywords:** lung squamous cell carcinoma, metastasis, prognosis, treatment pattern

## Abstract

Lung squamous cell carcinoma (LUSC) represents a minor proportion of nonsmall cell lung cancer (NSCLC) harboring a poor prognosis. Herein, retrospective medical record research was performed to investigate real‐world treatment patterns and identify the prognostic factors among LUSC patients. A total of 173 patients with a median age of 68 years were enrolled for analysis. Males were predominant (*n* = 143, 83%) and current or ex‐smokers contributed to 78% of the entire cohort. Pleura and lung were the most common metastatic sites, whereas brain metastasis was only 7%. After diagnosis, however, only 107 patients (62%) had received first‐line chemotherapy. In the chemotherapy cohort, median progression‐free survival (PFS) and overall survival (OS) were 3.9 and 11.1 months, respectively. After multivariable analysis, bone metastasis and the use of first‐line single‐agent chemotherapy independently predicted shorter PFS. For baseline characteristics, male sex, metastasis to lung, pleura, liver, and bone independently predicted worse OS. Regarding the treatment pattern, patients who had undergone standard first‐line doublet therapy and employed targeted therapies after disease progression linked to longer OS. In the real world, even those who underwent chemotherapy still had poor outcome. The findings may help clinicians to orchestrate the treatment strategies for LUSC patients and provide further direction of large‐scale studies.

## 1 INTRODUCTION

Lung cancer is currently the leading cause of cancer‐related deaths worldwide. The World Health Organization (WHO) reported approximately 2.2 million new lung cancer cases as well as 1.8 million deaths in 2020, contributing to a huge medical burden globally.^1^ For decades, cancer has been the major cause of death in Taiwan. Among all the malignancies, lung cancer accounts for the highest number of deaths, causing approximately 20% of all cancer‐related deaths in 2019.^2^

Lung squamous cell carcinoma (LUSC) is the second most common type of nonsmall cell lung cancer (NSCLC), accounting for 25%–30% of NSCLC cases.^3^ LUSC is distinct from lung adenocarcinoma (LUAD) for several aspects such as histopathological characteristics, risk factors, clinical features, treatment options, and prognosis. Generally, the patients of LUSC are more likely to be smokers and older.^4^ LUSCs tend to locate in the central part of the lungs, causing obstructive lung collapse, whereas LUADs are more often located in peripheral areas and usually present asymptomatically in the very beginning.

Targetable driver mutations such as EGFR, ALK, or ROS1 alterations are exclusively found in adenocarcinoma histology. Consequently, no effective targeting agents for LUSC have ever been identified, making pure LUSC a difficult‐to‐treat disease with poorer prognosis due to less‐effective treatment options during pre‐immunotherapy era.^5^ Therefore, based on the current National Comprehensive Cancer Network (NCCN) guideline, broad molecular testing is not recommended for the pure LUSC histology except for those in nonsmoking females or the diagnosis is established from small biopsy specimens.^6^ Immunotherapy has now played an important role in advanced LUSC. Those who receive immune checkpoint inhibitors experience an improvement of survival.^7^ Therefore, the combination of immunotherapy and chemotherapy is recommended as a first‐line treatment choice.^6^ However, the majority of LUSC patients in Taiwan do not receive immunotherapy because of economic issues, and chemotherapy alone is available in most clinical scenarios.

Prognostic factors that affect NSCLC are extensively studied. Various clinical factors have been identified as prognostic in different contexts, for example, age, sex and performance status, etc.^8^, ^9^ The process of metastasis is thought as nonrandom and therefore the metastatic pattern may also harbor a prognostic implication.^10^ However, fewer clinical studies investigate the prognostic factors focusing on LUSC. To improve the treatment efficacy of LUSC, this study aimed to investigate real‐world treatment patterns and explore prognostic factors of metastatic LUSC under chemotherapies. With a deeper understanding of LUSC, it is hoped more efficient management for treating LUSC patients can be determined.

## 2 METHODS

### 2.1 Study design and population (Figure 1)

**FIGURE 1:**
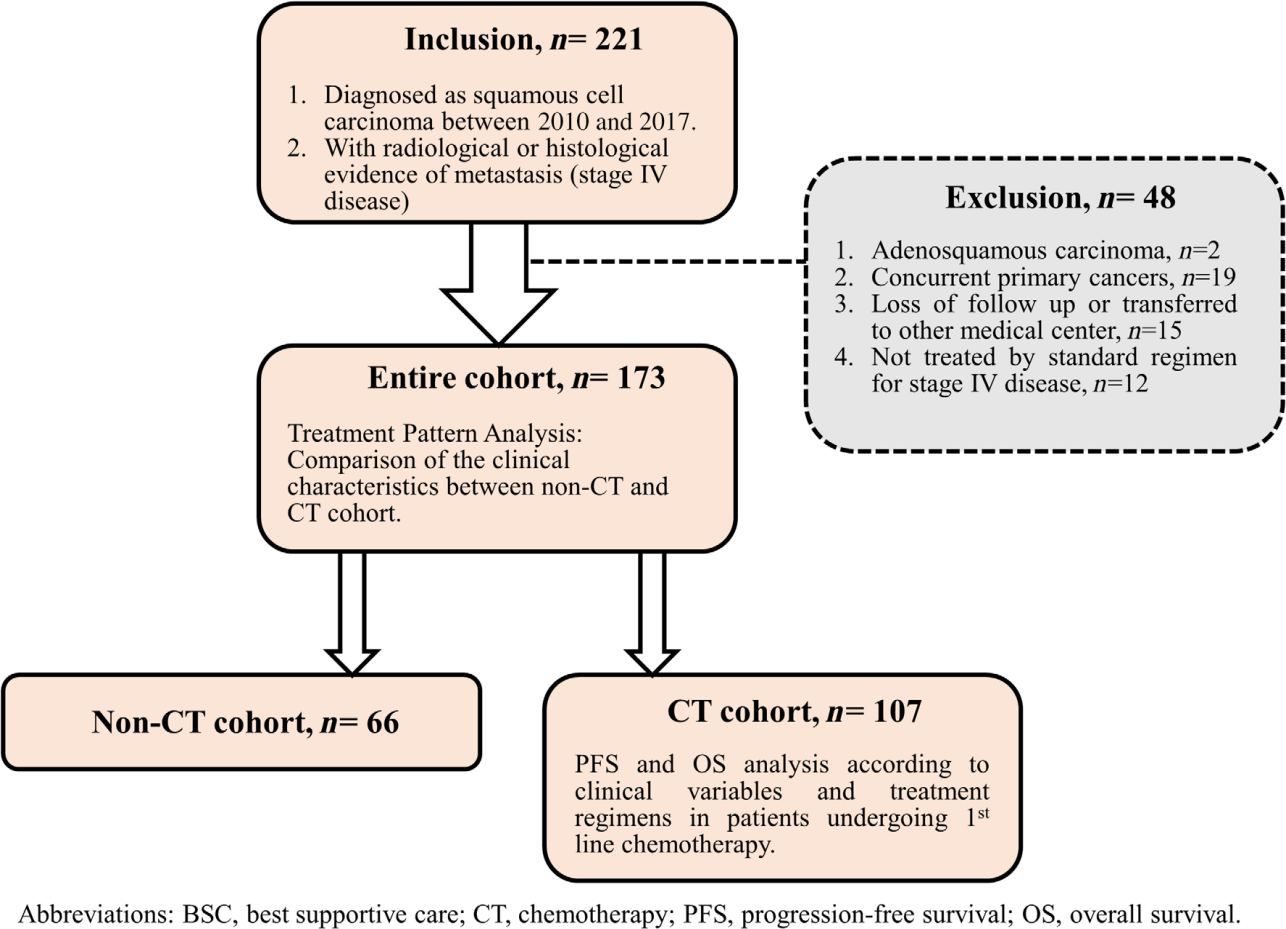
Flow chart of patient selection. BSC, best supportive care; CT, chemotherapy; PFS, progression‐free survival; OS, overall survival

The present study is a retrospective medical record review performed in two hospitals (one medical center and one community‐based hospital) in southern Taiwan. Patients confirmed as stage IV LUSC by AJCC 7th edition in the Cancer Registry Database of Kaohsiung Medical University Hospital (KMUH) and Kaohsiung Municipal Ta‐Tung Hospital (KMTTH) between 2010 and 2017 were enrolled; note that patients diagnosed after 2018 were not recruited because the new cancer staging system (AJCC 8th) was applied. Major exclusion criteria included those with adeno‐squamous carcinoma in histology, concomitant primary cancer of other organs, missing data or loss of follow‐up, and those without guideline‐based first‐line treatment. The study protocol was approved by the Institutional Review Board (IRB) of KMUH (KMUHIRB‐E(I)‐20200180).

### 2.2 Clinical data collection

Clinical demographic data including age, sex, smoking status, body mass index (BMI), performance status and co‐morbidities (CCI, Chalson's Comorbidity Index)^11^, ^12^ were recorded from the cancer registry database, and an electronic medical record review was performed to extract information concerning metastatic sites and their frequency of occurrence. Treatment modalities such as chemotherapies, including specific chemotherapeutic agents, radiation therapies, targeted therapies and surgery for the primary site were also obtained. Patients without definitive cancer treatment with chemotherapy were defined as the non‐CT (without chemotherapy) cohort, whereas those who underwent first‐line chemotherapy were defined as the CT cohort. In the non‐CT cohort, palliative radiation therapy for metastatic lesions was allowed. The primary outcomes were progression‐free survival (PFS) and overall survival (OS). PFS was defined as the periods from the start of chemotherapy to disease progression or death, depending on which comes first. OS was calculated as the duration from the beginning of chemotherapy to death.

### 2.3 Statistical analysis

Clinical variables such as age, sex, BMI, smoking status, performance status, CCI, metastatic sites and numbers were further stratified by patients receiving first‐line chemotherapy or not. The between‐group difference of categoric variables was tested by χ^2^ method or Fisher's exact test while continuous variables were tested by the Mann–Whitney U method.

PFS and OS were further analyzed with each clinical variable in the chemotherapy‐treated cohort with the difference of survival probability between the groups being examined by using the Kaplan–Meier method with the log‐rank test. Univariate and multivariable Cox proportional hazards regression analysis were used to identify the effect of different clinical features on PFS and OS. After the univariate analyses, all variables were included to construct a maximal model of multivariable analysis to assess the independent effect of the different variables. We also used backward variable selection method with the Akaike Information Criterion, removing those variables with p‐value more than 0.1, to develop a reduced multivariable model. For analyzing the prognostic effect of targeted therapy after disease progression to first‐line chemotherapy, a propensity score matching (PSM) model was established. The model was constructed by 1:2 comparable parts that were stratified by using targeted agents or not, and adjusted for age, sex, Eastern Cooperative Oncology Group (ECOG), smoking history and the number of metastasis. The caliper width for the model is 0.2 of the standard deviation of the logit of the propensity score. The p‐value less than 0.05 was set as a significant level. Data were analyzed using R language (version 4.1.0).

## 3 RESULTS

### 3.1 Patient characteristics

Between 2010 and 2017, a total of 221 newly diagnosed stage IV LUSC patients were recorded in the Cancer Registry Database in KMUH and KMTTH. After excluding those with mixed adeno‐squamous histology (*n* = 2), concomitant primary cancers in other organs (*n* = 19), missing data or loss of follow‐up (*n* = 15) and treatment not confined in guideline‐recommended regimens for stage IV NSCLC (*n* = 12), 173 patients were retained for further analysis (Figure 1).

The median age of those patients was 68 with the interquartile range (IQR) of 61–76.5. Males were the predominant population (*n* = 143, 83%) while current and/or ex‐smokers contributed to 78% of the entire cohort. One hundred and thirty‐five patients (78%) had BMI less than 25 while 38 (22%) were overweight (BMI equal to or more than 25 based on WHO definition). Upon diagnosis, 121 (70%) patients were at good performance status (ECOG 0 or 1).

Compared with the CT cohort, patients in non‐CT cohort were statistically older (median age 75 vs. 66, p < 0.001) and had worse baseline performance status (48% vs. 19% in the group of ECOG ≥2, p < 0.001). Other clinical variables, such as sex, smoking history, BMI, tumor‐node‐metastas stages and number of metastatic sites were similar between those taking chemotherapy or not; however, statistically significant difference was noted in the pattern of metastasis. Compared to the non‐CT cohort, the CT cohort had more patients with bone metastasis (34% vs 18%, p = 0.042). The full baseline characteristics upon diagnosis of these patients are summarized in Table 1.

**TABLE 1:** Baseline characteristics in non‐CT and CT cohorts

Variables	All patients	Non‐CT cohort	CT cohort	p Value
Number (%)	173 (100)	66 (38%)	107 (62%)	
Age – median (IQR)	68 (61‐76.5)	75 (65‐82)	66 (60‐73)	<0.001^a^
Age—*n* (%)				
< 65 years old	61 (36%)	15 (23%)	47 (44%)	0.004
≥65 years old	111 (64%)	51 (77%)	60 (56%)	
Sex—*n* (%)				
Female	30 (17%)	9 (14%)	21 (20%)	0.272
Male	143 (83%)	57 (86%)	86 (80%)	
Smoking history – *n* (%)				
Never smoker	38 (22%)	15 (23%)	23 (21%)	0.975
Ex‐ or current smoker	135 (78%)	51 (77%)	84 (79%)	
BMI				
Mean ± SD		21.9±3.6	22.9±3.4	0.060^a^
BMI <25 – *n* (%)	135 (78%)	53 (65%)	82 (77%)	0.615
Overweight – *n* (%)	38 (22%)	13 (20%)	25 (23%)	
Performance status – *n* (%)				
ECOG = 0 or 1	121 (70%)	34 (52%)	87 (81%)	<0.001
ECOG ≥ 2	52 (30%)	32 (48%)	20 (19%)	
Comorbidity				
CCI < 10 76 (44%)	19 (29%)	57 (53%)	0.002	
CCI ≥ 10	97 (56%)	47 (71%)	50 (47%)	
T stage – *n* (%)				
T1	3 (2%)	0 (0%)	3 (3%)	0.382
T2	25(14%)	8 (12%)	17 (16%)	
T3	34 (20%)	14 (21%)	20 (19%)	
T4	111 (64%)	44 (67%)	67 (62%)	
N stage – *n* (%)				
N0	27 (15%)	11 (17%)	16 (15%)	0.189
N1	10 (6%)	2 (3%)	8 (8%)	
N2	63 (37%)	20 (30%)	43 (40%)	
N3	73 (42%)	33 (50%)	40 (37%)	
M stage – *n* (%)				
M1a	89 (51%)	37 (56%)	52 (49%)	0.284
M1b	84 (49%)	29 (44%)	55 (51%)	
Number of metastatic sites				
1	79 (46%)	27 (41%)	52 (49%)	0.321
≥2	94 (54%)	39 (59%)	55 (51%)	
Metastatic sites – *n* (%)				
Lung	63 (36%)	25 (38%)	38 (36%)	0.780
Pleura or pleural effusion	115 (66%)	48 (73%)	67 (62%)	0.161
Pericardium	32 (18%)	17 (26%)	15 (14%)	0.084
Brain	13 (7%)	4 (6%)	9 (8%)	0.591
Liver	23 (13%)	10 (15%)	13 (12%)	0.667
Bone	48 (28%)	12 (18%)	36 (34%)	0.042
Adrenal	19 (11%)	6 (9%)	13 (12%)	0.559
Others	9 (5%)	3 (4.5%)	6 (6%)	0.604

^a^ The p value of the numerical variables were tested by Mann–Whitney U method. In other comparisons between categorical variables, the p value was tested by the *χ* ^2^ method or the Fisher's exact test.

Abbreviations: BMI, body mass index; CCI, Charlson comorbidity index; CT, chemotherapy; ECOG, Eastern Cooperative Oncology Group; IQR, interquartile range; SD, standard deviation.

### 3.2 Status of metastasis

Based on the inclusion criteria, all patients had at least one metastatic site. Among those, 84 (49%) patients had extra‐thoracic metastasis, and 94 (54%) patients had 2 or more metastatic sites. Pleura (66%) was the most common metastatic site, followed by lung (36%), bone (28%), pericardium or pericardial effusion (18%), liver (13%), and adrenal glands (11%). Brain metastasis was relatively infrequent with only 7% of cases among this study group.

### 3.3 Treatment patterns

In this real‐world data (Table 1), 66 patients (38%) chose the supportive care (non‐CT) without having any chemotherapeutic agent. On the contrary, 107 patients (62%) had their first‐line chemotherapies with either single agent or standard platinum‐doublets based on the physicians' decision.

Among those who underwent first‐line chemotherapy, 33 patients (31%) received single chemotherapeutic agent whereas 74 (69%) underwent standard platinum‐based doublets. Different cytotoxic chemo‐agents were used in combination with platinum such as gemcitabine, docetaxel and vinorelbine according to the physicians' and the patients' preferences. In the CT cohort, 38 patients (36%) took gemcitabine, 41 (38%) took docetaxel, and 28 (26%) were given vinorelbine.

Nine patients received surgery for primary tumors prior to chemotherapy. The operations were performed for those with difficulty on tissue proof or with presumed early stages but later been confirmed as stage IV diseases after surgery. Concurrent radiation therapy for the primary site was applied in 19 (18%) patients undergoing first‐line systemic chemotherapy. After disease progression, there were another 14 patients receiving radiation alone or in combination with later‐line chemotherapy to achieve better disease control for primary lung tumors. Besides, 22 patients (21%) had targeted therapy with tyrosine kinase inhibitors after disease progression on chemotherapy.

### 3.4 Progression‐free survival

**FIGURE 2:**
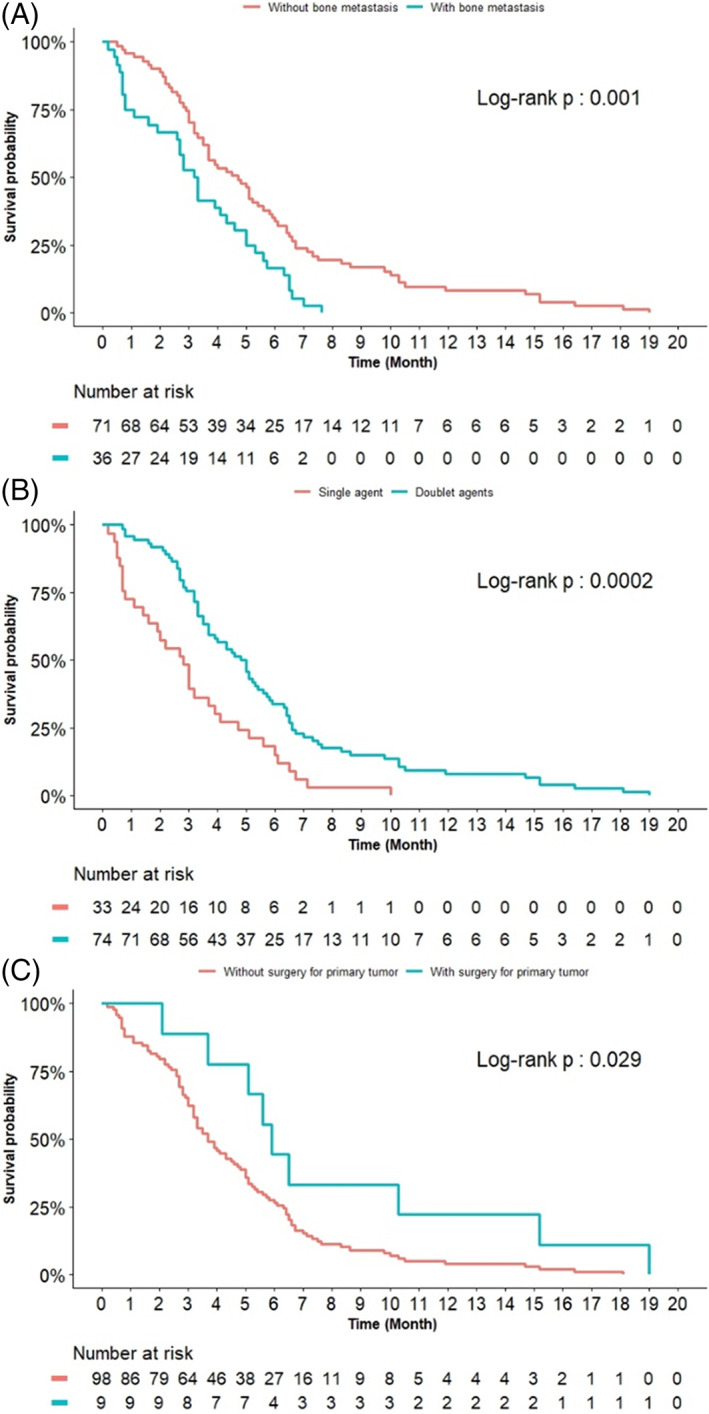
Progression‐free survival of stage IV LUSC patients undergoing first‐line chemotherapy, stratified by the presence of bone metastasis (A), the employment of single or doublet therapy (B), and the history of surgical resection of the primary tumor (C)

The median PFS was 3.9 (IQR: 2.9–4.9) months among the patients receiving first‐line chemotherapy (Table 2). Regarding the baseline characteristics, the PFS was not different by age, sex, smoking history, BMI, performance status and comorbidity, although more metastatic sites (*n* ≥ 2) and extra‐thoracic metastasis (M1b) were associated with shorter PFS. Bone metastasis was substantially linked to shorter PFS compared with those without bone involvement (3.2 vs. 4.7 months, p = 0.001, Figure 2A).

**TABLE 2:** Progression‐free and overall survival analysis in CT cohort

Variables	*n* (%)	PFS (m)	IQR	p	OS (m)	IQR	p
All patients	107 (100%)	3.9	(2.9–4.9)		11.1	(6.8–19.5)	
Age – *n* (%)				0.507			0.015
< 65 years old	47 (44%)	4.6	(3.0–6.5)		12.2	(7.6–23.7)	
≥ 65 years old	60 (56%)	3.7	(2.3–5.9)		10.1	(5.8–18.2)	
Sex – *n* (%)				0.786			0.084
Female	21 (20%)	5.0	(2.9–5.3)		18.8	(6.8–26.0)	
Male	86 (80%)	3.7	(2.6–6.5)		10.1	(7.1–17.7)	
Smoking history – *n* (%)				0.872			0.211
Never smoker	23 (21%)	5.0	(1.4–6.4)		14.7	(4.8–26.0)	
Ex– or current smoker 84 (79%)		3.7	(2.7–6.4)		10.1	(7.1–17.7)	
BMI				0.424			0.065
BMI <25 – *n* (%)	82 (77%)	3.9	(2.6–6.1)		9.6	(6.6–18.2)	
Overweight – *n* (%)	25 (23%)	5.0	(2.7–6.6)		15.4	(10.1– 13.2)	
Performance status – *n* (%)				0.459			0.015
ECOG = 0 or 1	87 (81%)	4.3	(2.7–6.4)		12.2	(7.1–20.8)	
ECOG ≥ 2	20 (19%)	3.3	(1.7–6.4)		8.0	(4.8–12.6)	
Comorbidity				0.790			0.204
CCI<10	57 (53%)	4.5	(3.2–5.9)		12.2	(8.1–16.3)	
CCI ≥ 10	50 (47%)	3.7	(2.9–4.4)		11.0	(8.3–13.7)	
T stage – *n* (%)				0.195			0.042
T1	3 (2%)	3.0	(0.7–6.3)		11.0	(3.1–52.7)	
T2	17 (16%)	3.3	(2.2–5.1)		11.4	(7.7–16.9)	
T3	20 (19%)	5.4	(3.2–6.5)		15.6	(8.0–23.7)	
T4	67 (63%)	4.0	(2.6–6.4)		9.6	(5.6–18.8)	
N stage – *n* (%)				0.417			0.062
N0	16 (15%)	3.7	(1.7–7.3)		14.0	(7.7–52.7)	
N1	8 (8%)	3.5	(0.7–5.1)		8.0	(6.0–11.4)	
N2	43 (40%)	3.7	(2.7–5.6)		13.0	(7.8–19.8)	
N3	40 (37%)	3.9	(2.2–6.5)		9.6	(5.0–16.5)	
M stage – *n* (%)				0.017			0.302
M1a	52 (49%)	4.8	(2.8–6.6)		14.1	(8.0–19.2)	
M1b	55 (51%)	3.5	(1.9–5.6)		8.5	(4.8–19.6)	
Number of metastatic sites				0.015			0.009
1	52 (49%)	4.5	(2.7–7.3)		14.3	(8.0–21.7)	
≥2	55 (51%)	3.7	(2.3–5.9)		8.1	(4.8–15.7)	
Metastatic sites – *n* (%)							
Lung				0.556			0.230
Yes	38 (36%)	3.9	(2.3–6.1)		9.6	(6.6–19.2)	
No	69 (64%)	3.7	(2.7–6.5)		11.9	(7.6–20.2)	
Pleura or pleural effusion				0.138			0.878
Yes	67 (63%)	4.3	(2.7–6.6)		10.0	(6.5–19.8)	
No	40 (37%)	3.3	(2.2–5.6)		11.1	(6.8–19.2)	
Pericardium				0.624			0.047
Yes	15 (14%)	4.7	(3.0–6.4)		9.6	(5.6–13.9)	
No	92 (86%)	3.7	(2.4–6.4)		12.2	(6.8–20.8)	
Brain				0.272			0.131
Yes	9 (8%)	3.3	(1.9–3.9)		7.6	(3.8–8.1)	
No	98 (92%)	4.1	(2.7–6.5)		12.2	(7.2–19.8)	
Liver				0.093			0.903
Yes	13 (12%)	2.7	(1.6–5.1)		6.0	(3.8–20.8)	
No	94 (88%)	4.0	(2.7–6.5)		11.8	(7.6–19.5)	
Bone				0.001			0.122
Yes	36 (34%)	3.2	(0.8–5.0)		8.0	(3.8–15.7)	
No	71 (66%)	4.7	(2.9–6.7)		13.9	(7.8–19.8)	
Adrenal				0.273			0.550
Yes	13 (12%)	3.3	(2.7–5.3)		13.0	(7.2–16.9)	
No	94 (88%)	4.0	(2.6–6.5)		11.1	(6.8–19.6)	
Others				0.040			0.254
Yes	6 (6%)	2.9	(2.6–3.3)		7.6	(3.8–8.0)	
No	101 (94%)	4.1	(2.7–6.5)		11.8	(6.8–19.6)	
Treatment							
Chemotherapy				<0.001			0.001
Single	33 (31%)	2.8	(0.8–4.7)		7.9	(3.8–12.6)	
Doublet	74 (69%)	4.8	(3.2–6.6)		14.6	(7.8–21.8)	
Regimens				0.666			0.960
Gem–based	38 (36%)	4.3	(2.7–6.4)		10.1	(7.6–19.6)	
Doc–based	41 (38%)	4	(2.6–6.5)		11.8	(4.7–21.8)	
Vin–based	28 (26%)	3.3	(2.2–5.2)		11.0	(5.6–15.7)	
Surgery of primary tumor				0.029			0.027
Yes	9 (8%)	5.9 (5.1–10.3)		16.8	(13.0–23.7)		
No	98 (92%)	3.7	(2.6–6.3)		9.6	(6.5–19.2)	
Radiation therapy				0.426			0.931
Yes	19 (18%)^a^; 33 (31%)^b^	3.0	(2.2–6.5)		13.0	(8.2–19.8)	
No	88 (82%)^a^; 74 (69%)^b^	4.0	(2.7–6.4)		9.6	(5.8–19.5)	
Targeted therapy							0.059
Yes	22 (21%)				16.9	(9.6–23.7)	
No	85 (79%)				9.6	(5.6–17.7)	

*Note*: All p values were tested by Kaplan–Meier method and log‐rank test.

Abbreviations: BMI, body mass index; CCI, Charlson comorbidity index; CT, chemotherapy; Doc‐based, docetaxel‐based regimen; ECOG, Eastern Cooperative Oncology Group; Gem‐based, gemcitabine‐based regimen; IQR, interquartile range; Vin‐based, vinorelbine‐based regimen.

^a^ The case number with radiation on primary tumor during first‐line treatment which was calculated for progression‐free survival.

^b^ The patients underwent radiation for primary tumor in the full course which were analyzed for the overall survival.

Patients taking standard doublets chemotherapy had significantly better PFS than patients taking single agents (4.8 vs. 2.8 months, p < 0.001, Figure 2B), although no specific chemotherapeutic agent was found to demonstrate better PFS versus other agents, either being used alone or in combination with platinum. Additionally, those who had prior surgery on their primary tumor had longer PFS of their first‐line treatment (Figure 2C).

### 3.5 Overall survival

Median OS was 11.1 (IQR: 6.8–19.5) months among the patients receiving first‐line chemotherapy (Table 2), differing significantly by age and performance status. Those aged less than 65 years had longer OS compared to those who were older (12.2 vs. 10.1 months, p = 0.015). Better performance status also conferred longer survival (12.2 vs. 8.0 months, p = 0.015). In the Kaplan–Meier survival analysis, female gender and overweight contributed to better OS, though nonsignificantly.

In terms of the metastatic pattern, those with 2 or more metastatic sites harbored shorter OS compared with those with only one metastatic site (8.1 vs. 14.3 months, p = 0.009). Among various metastatic sites, pericardial metastasis exhibited a trend toward shorter OS (9.6 vs. 12.2 months, p = 0.047).

**FIGURE 3:**
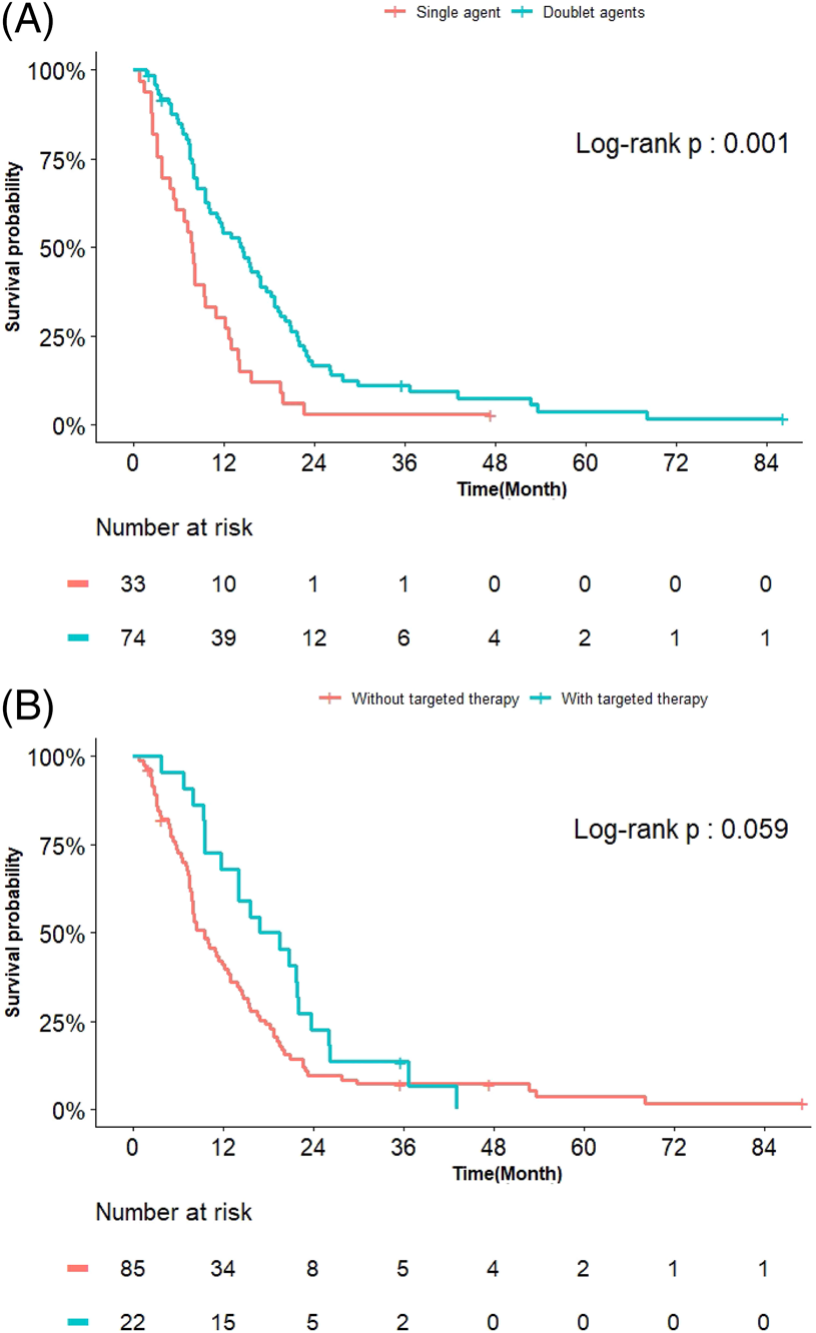
Overall survival of stage IV lung squamous cell carcinoma patients undergoing first‐line chemotherapy, stratified by the employment of single or doublet therapy (A), and the use of postprogression targeted therapy (B)

Patients taking standard doublets survived longer than those with single agent (14.6 vs. 7.9 months, p = 0.001, Figure 3A), although the OS did not differ across whatever chemotherapeutic agents used in the platinum‐doublets by Kaplan–Meier analysis. Additionally, those who had prior surgery on a primary lung tumor also had longer OS (16.8 vs. 9.6 months, p = 0.027). Targeted therapy used after progression was also linked to better OS (16.9 vs. 9.6 months, p = 0.059, Figure 3B), though was not statistically significant.

### 3.6 Multivariable analysis of PFS and OS

In both maximum and backward selection models of multivariable regression, bone metastasis is an independent predicting factor for shorter PFS (HR:2.57, CI:1.63–4.05, p < 0.001), whereas using standard platinum‐based doublet confers better PFS (HR:0.40, CI: 0.26–0.62, p < 0.001, Table 3).

**TABLE 3:** Cox proportional regression analyses for progression‐free survival

	Univariate			Maximal model			Reduced model (backward model)		
Variable	HR	95% CI	p Value	HR	95% CI	p Value	HR	95% CI	p‐value
Sex (male vs. female)	**1.07**	(0.66–1.74)	0.775	**1.46**	(0.62–3.45)	0.382			
Age (>65 vs. <65)	**1.14**	(0.77–1.68)	0.511	**1.48**	(0.82–2.67)	0.191			
BMI (>25 vs. <25)	**0.83**	(0.52–1.31)	0.415	**1.23**	(0.71–2.12)	0.452			
Smoker versus nonsmoker	**0.97**	(0.61–1.54)	0.879	**0.61**	(0.28–1.36)	0.231			
ECOG ≥2 versus <2	**1.20**	(0.74–1.97)	0.461	**0.96**	(0.53–1.73)	0.885			
CCI>=10 versus <10	**1.05**	(0.72–1.55)	0.788	**1.09**	(0.62–1.89)	0.770			
T2 versus T1	**0.79**	(0.23–2.72)	0.711	**0.63**	(0.15–2.73)	0.541			
T3 versus T1	**0.41**	(0.12–1.40)	0.154	**0.50**	(0.11–2.29)	0.372			
T4 versus T1	**0.62**	(0.20–2.00)	0.426	**0.71**	(0.17–2.97)	0.641			
N1 versus N0	**1.97**	(0.82–4.72)	0.129	**1.34**	(0.49–3.65)	0.573			
N2 versus N0	**1.51**	(0.83–2.74)	0.174	**1.47**	(0.63–3.46)	0.376			
N3 versus N0	**1.48**	(0.80–2.72)	0.208	**0.83**	(0.34–2.05)	0.684			
M1b versus M1a	**1.61**	(1.09–2.38)	**0.018**	**1.07**	(0.51–2.25)	0.861			
Metastatic site >1 versus 1	**1.64**	(1.10–2.45)	**0.015**	**1.32**	(0.63–2.77)	0.469			
Lung to lung metastasis	**1.12**	(0.75–1.67)	0.571	**1.09**	(0.56–2.13)	0.805			
Pleural metastasis	**0.75**	(0.50–1.11)	0.146	**0.93**	(0.47–1.85)	0.842			
Pericardium metastasis	**1.15**	(0.66–2.00)	0.632	**0.52**	(0.26–1.07)	0.076			
Brain metastasis	**1.47**	(0.74–2.93)	0.271	**1.64**	(0.68–3.96)	0.274			
Liver metastasis	**1.66**	(0.92–2.99)	0.090	**1.71**	(0.76–3.88)	0.200	**1.78**	(0.98–3.24)	0.058
Bone metastasis	**2.01**	(1.31–3.07)	**0.001**	**2.95**	(1.46–5.96)	**0.003**	**2.57**	(1.63–4.05)	**<0.001**
Adrenal metastasis	**1.37**	(0.76–2.47)	0.289	**0.72**	(0.31–1.68)	0.447			
Treatment									
Doublet versus single	**0.46**	(0.30–0.70)	**<0.001**	**0.26**	(0.13–0.50)	**<0.001**	**0.40**	(0.26–0.62)	**<0.001**
Docetaxel‐ versus gemcitabine‐based	**1.20**	(0.77–1.87)	0.430	**0.87**	(0.51–1.49)	0.609			
Vinorelbine‐ versus gemcitabine‐based	**1.20**	(0.73–1.98)	0.464	**0.65**	(0.33–1.26)	0.199			
Surgery for primary tumor	**0.46**	(0.22–0.95)	**0.036**	**0.52**	(0.20–1.36)	0.183	**0.49**	(0.23–1.02)	0.055
Radiation for primary tumor	**1.24**	(0.75–2.04)	0.409	**1.92**	(0.99–3.70)	0.053			

Abbreviations: BMI, body mass index; CCI, Charlson comorbidity index; ECOG, Eastern Cooperative Oncology Group.

In the backward selection‐reduced model, male sex, metastasis to lung, pleura, liver, and bone demonstrated independent predicting ability for poor OS (Table 4). In contrast, taking standard platinum‐based doublet contributed to longer OS (HR: 0.31, CI:0.19–0.53, p < 0.001). Besides, we found that there was slight OS benefit of docetaxel‐ and vinorelbine‐containing regimens instead of gemcitabine‐containing regimen after the multivariable analysis. Moreover, targeted therapy used after progression on chemotherapy predict better OS according to the results (HR 0.33, CI 0.19–0.59, p < 0.001).

**TABLE 4:** Cox proportional regression analyses for overall survival

	Univariates			Maximal model			Reduced model (backward model)		
Variable	HR	95% CI	p Value	HR	95% CI	p Value	HR	95% CI	p‐value
Sex (male vs. female)	**1.57**	(0.94–2.64)	0.087	**3.86**	(1.52–9.79)	**0.005**	**2.59**	(1.42–4.72)	**0.002**
Age (>65 vs. <65)	**1.67**	(1.10–2.55)	**0.017**	**1.29**	(0.73–2.26)	0.378			
BMI (>25 vs. <25)	**0.64**	(0.40–1.03)	0.066	**0.64**	(0.37–1.13)	0.125			
Smoker versus non‐smoker	**1.36**	(0.84–2.22)	0.214	**0.75**	(0.31–1.80)	0.521			
ECOG ≥2 versus <2	**1.89**	(1.12–3.19)	**0.018**	**1.37**	(0.74–2.54)	0.315			
CCI ≥10 versus <10	**1.29**	(0.87–1.93)	0.208	**0.73**	(0.42–1.26)	0.254			
T2 versus T1	**1.42**	(0.40–5.03)	0.582	**1.25**	(0.27–5.84)	0.778			
T3 versus T1	**0.76**	(0.22–2.62)	0.662	**0.49**	(0.10–2.46)	0.386			
T4 versus T1	**1.69**	(0.51–5.59)	0.386	**1.28**	(0.26–6.31)	0.764			
N1 versus N0	**2.79**	(1.12–6.97)	**0.028**	**2.40**	(0.74–7.80)	0.145			
N2 versus N0	**1.54**	(0.81–2.96)	0.190	**1.79**	(0.68–4.72)	0.240			
N3 versus N0	**2.10**	(1.09–4.06)	**0.027**	**1.76**	(0.63–4.95)	0.284			
M1b versus M1a	**1.23**	(0.83–1.82)	0.302	**1.12**	(0.54–2.31)	0.757			
Metastatic site >1 versus 1	**1.71**	(1.14–2.55)	**0.009**	**1.16**	(0.55–2.47)	0.699			
Lung to lung metastasis	**1.28**	(0.85–1.94)	0.236	**1.76**	(0.85–3.67)	0.130	**1.80**	(1.09–2.97)	**0.023**
Pleural metastasis	**0.97**	(0.65–1.45)	0.869	**1.99**	(0.96–4.12)	0.066	**2.06**	(1.04–2.89)	**0.009**
Pericardium metastasis	**1.76**	(1.00–3.10)	0.051	**0.83**	(0.40–1.71)	0.609			
Brain metastasis	**1.73**	(0.83–3.59)	0.142	**2.00**	(0.69–5.77)	0.199			
Liver metastasis	**1.04**	(0.58–1.88)	0.898	**2.02**	(0.83–4.94)	0.121	**2.18**	(1.09–4.33)	**0.027**
Bone metastasis	**1.38**	(0.92–2.08)	0.123	**2.38**	(1.12–5.06)	**0.024**	**2.91**	(1.73–4.88)	<**0.001**
Adrenal metastasis	**1.20**	(0.67–2.15)	0.550	**0.67**	(0.29–1.57)	0.362			
Doublet versus single	**0.47**	(0.31–0.73)	**0.001**	**0.36**	(0.18–0.70)	**0.003**	**0.31**	(0.19–0.53)	<**0.001**
Docetaxel‐ versus gemcitabine‐based	**0.94**	(0.59–1.49)	0.780	**0.46**	(0.26–0.83)	**0.009**	**0.57**	(0.34–0.96)	**0.034**
Vinorelbine‐ versus gemcitabine‐based	**0.95**	(0.57–1.59)	0.849	**0.53**	(0.27–1.07)	0.078	**0.51**	(0.28–0.93)	**0.029**
Surgery for primary tumor	**0.43**	(0.20–0.93)	**0.032**	**0.82**	(0.31–2.16)	0.693			
Radiation for primary tumor	**1.02**	(0.67–1.55)	0.940	**1.93**	(1.12–3.33)	**0.018**			
Targeted therapy after progression	**0.63**	(0.39–1.02)	0.062	**0.30**	(0.15–0.60)	**0.001**	**0.33**	(0.19–0.59)	<**0.001**

Abbreviations: BMI, body mass index; CCI, Charlson comorbidity index; ECOG, Eastern Cooperative Oncology Group.

## 4 DISCUSSION

This real‐world retrospective study demonstrated the egregious outcome of stage IV LUSC patients. A large proportion of patients (non‐CT cohort, *n* = 66, 38%) took supportive care upon diagnosis due to their old age or poor physical status. Even in those receiving first‐line chemotherapy (CT cohort), about 31% of patients simply used single chemotherapeutic agent instead of standard platinum‐doublet regimens. The median PFS was only 3.9 months and OS 11.1 months in the CT cohort. Among the various baseline clinical characteristics, bony metastasis predicted shorter PFS and OS independently. Regarding the treatment pattern, we found that standard platinum‐based doublet was strongly associated with better PFS and OS. In addition, targeted therapy used as subsequent systemic therapy was linked to better OS.

To our surprise, a large proportion of patients did not agree to chemotherapy and chose the best supportive care in our study. In a previous investigation in Canada, a much larger cohort of stage IV LUSC (*n* = 2056) was recruited between 2010 to 2015. The authors reported only 473 patients (23.1%) received first‐line chemotherapy, including chemotherapy alone or chemoradiation therapy. Among those undergoing chemotherapy, cisplatin or carboplatin with gemcitabine were employed in half of the patients.^13^ In our facilities, in contrast, the docetaxel‐based regimen is used most often (*n* = 41, 38%) followed by gemcitabine (*n* = 38, 36%) and vinorelbine (*n* = 28, 26%).

The low rate of patients receiving systemic chemotherapy could be explained by physical conditions and socioeconomic status. In our investigation, those with older age and poorer physical status were likely to choose the best supportive care. LUSC patients, who are highly related to cigarette smoking, would correlate to more comorbidities and lower cardiopulmonary reserve.^14^ Moreover, cigarette smoke is also associated with lower socioeconomic status in epidemiologic studies.^15^ Compared to patients with other histologies of NSCLC, LUSC patients utilize more health care resources and have lower income status.^13^ Moreover, patients with low socioeconomic status are prone to choose more conservative management and are linked to the discordance of guideline‐recommended therapies.^16^ Though our study did not retrieve socio‐economic data from individual patients, we believe that socioeconomic level might play an important role in altering patients' or their surrogates' medical decision‐making.

BMI, a surrogate of nutritional status, has been regarded as a prognostic factor in both localized^8^ and advanced lung cancer.^17^ In these reports, overweight is associated with better survival. In our study, however, the OS effect of overweight is numerically better (HR 0.64) but statistically nonsignificant (p‐value 0.066) in the univariate analysis. The possible explanations may include the confounding effect of other clinical variables that also influence BMI, such as age, ECOG, and CCI. After competing with these factors in multivariable analysis, the BMI seems to be less relevant to the OS. Another concern is the cutoff value of BMI. Some studies demonstrated the OS benefit in those with BMI > 30, which is different from our setting.^18^ Though the survival benefit of overweight on OS is not statistically significant in our study, we still emphasize the detrimental effect of cachexia in cancer patients and remind clinicians to pay more attention on the patients' nutritional status.

In view of cancer biology, metastasis is not a randomly occurring process. From the old “seed and soil” to the latest “pre‐metastatic niche” theories, cancer cells exhibit delicate and harmonic mechanisms to metastasize and colonize into a specific organ.^19^ Moreover, metastasis to the various organs can infer different outcomes, and a considerable amount of literature has demonstrated the prognostic implications of the metastatic site—especially in LUAD.^20^, ^21^ In contrast, reports regarding the prognostic role of metastatic sites in LUSC are scarce. Gao et al. reported liver metastasis as the poor prognostic factor in 350 unresectable LUSC patients in China.^22^ However, bone metastasis was identified as an independent poor prognostic factor of both PFS and OS in our study. Larger population‐based studies are needed to confirm our findings, and molecular research could also be conducted to investigate the underlying mechanisms.

In the CT cohort, people who received doublet regimen as their first‐line chemotherapy had better survival than patients taking the single regimen in terms of PFS and OS. In clinical practice, clinicians tend to execute standard doublet therapy and reserve docetaxel for those of younger age or better physical status. However, the bias may be corrected by multivariable regression. Based on findings of this study, clinicians should consider standard doublet chemotherapy for those stage IV LUSC patients with younger age or adequate performance status. We observed better OS in those using docetaxel‐ or vinorelbine‐containing chemotherapies than gemcitabine. Because gemcitabine was reported to be associated with higher interstitial pneumonitis risk if combined with radiation therapy, the confounding effect from radiation therapy was theoretically considered. However, in our cohort, there was no inequality existed between the subgroups who were exposed to different chemo‐regimens. Therefore, the inferior survival effect of gemcitabine was not explained by the use the radiation therapy.

In our study, patients received targeted therapy after progression on first‐line chemotherapy harbored better OS. Previously, the beneficial effect of targeted agents, especially erlotinib, as a second‐line treatment for LUSC is reported in the literature. Because EGFR TKI, especially erlotinib (Tarceva) is indicated for the second‐line treatment or beyond after the failure of previous chemotherapy for NSCLC (not limited to adenocarcinoma).^23^, ^24^ Recently, a real‐world study in China exhibit survival benefit with first generation EGFR‐TKI in LUSC harboring EGFR mutation whether as first‐line treatment or beyond.^25^ In the final analysis of LUX‐Lung‐8 trial, both afatinib and erlotinib showed survival benefit and some of the patient being survived after more than 3 years.^26^ In addition, EGFR TKI is a well‐tolerated and available treatment option. Therefore, the patients who did not use targeted therapy may also have a worse performance status so that the presumed tolerable agent could not be given to them. To avoid the confounding effect, we performed a PSM model to construct 1:2 comparable groups of patients with or without targeted therapy (Table S1). After the analysis, the use of targeted therapy is still OS beneficial with p = 0.031 (Figure S1).

There are several limitations to our study. Firstly, the information about patient characteristics that influence the treatment decision including comorbidities and socioeconomic status, were not fully extractable from medical records. Secondly, the OS of our study might not represent OS nowadays. Although patients taking targeted therapy were analyzed in this study, the role of immunotherapy in LUSC was not demonstrated. Since immunotherapy for lung cancer during the study period was not common, no patients in our study ever took immunotherapy. From recent clinical studies, immunotherapy alone or in combination with chemotherapy benefit PFS and OS in certain group of LUSC patients. Third, we failed to demonstrate enough evidence to explain the inferior effect of gemcitabine‐containing regimen. Finally, the small sample size might result in difference not being statistically significant. Regardless, LUSC per se is a relatively minor proportion in lung cancer, and only stage IV patients were collected for analysis. Further analysis using national databases might help to solve this problem.

## 5 CONCLUSION

Our study demonstrated real‐world experience of how patients with metastatic LUSC were treated, which is less commonly emphasized in previous studies. More than one‐third of stage IV LUSC patients did not receive any kind of chemotherapeutic agent. Even in those who ever underwent first‐line chemotherapy, the prognosis remains poor. The employment of standard platinum‐based doublet as first‐line treatment confers better PFS and OS. In contrast, we identified bone metastasis as a strong prognostic factor contributing to shorter PFS and OS, though the molecular mechanism is not clear and basic research is further needed.

## CONFLICT OF INTEREST

All authors declare no conflict of interest.

## Supporting information


**Figure S1** Survival curve of LUSC patients with or without targeted therapy after propensity score matching.

**Table S1** Baseline characteristics of the unmatched and matched groups
