# Supplementary figures and images for: Real‐world treatment pattern and prognostic factors of stage IV lung squamous cell carcinoma patients
Source: Kaohsiung J Med Sci. 2022 Oct 10;38(10):1001–11. doi: 10.1002/kjm2.12599 (PMC11896242; doi:10.1002/kjm2.12599)

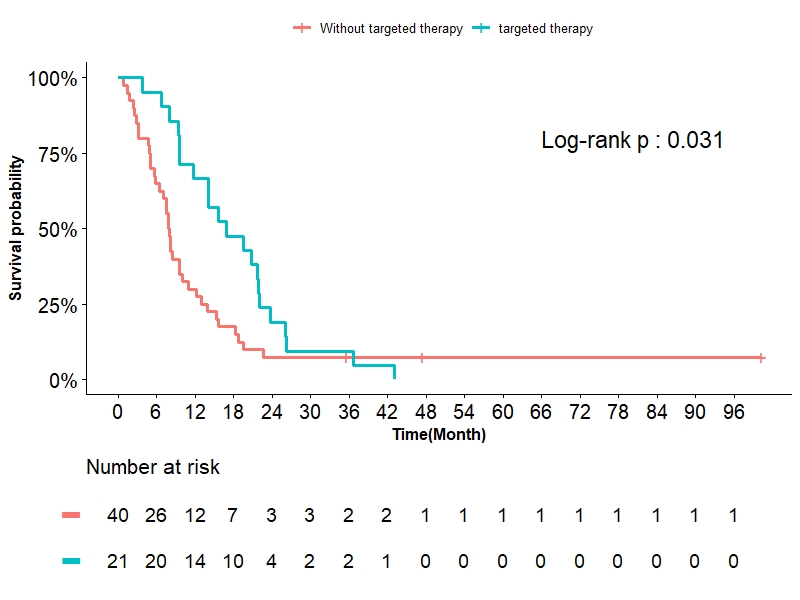

Supplement: Supplementary file 1 — Figure S1 Survival curve of LUSC patients with or without targeted therapy after propensity score matching. [file KJM2-38-1001-s002.jpeg]
