# Supplementary material for: Real‐world treatment pattern and prognostic factors of stage IV lung squamous cell carcinoma patients
Source: Kaohsiung J Med Sci. 2022 Oct 10;38(10):1001–11. doi: 10.1002/kjm2.12599 (PMC11896242; doi:10.1002/kjm2.12599)
Supplement: Supplementary file 2 — Table S1 Baseline characteristics of the unmatched and matched groups [file KJM2-38-1001-s001.docx]

| **Supplementary Table 1. Baseline characteristics of the unmatched and matched groups** | | | | | | |
| --- | --- | --- | --- | --- | --- | --- |
|  | **Before Propensity Score Matching** |  |  | **After Propensity Score Matching** |  |  |
|  | **Without targeted therapy** | **With targeted therapy** | ***p* value^*^** | **Without targeted therapy** | **With targeted therapy** | ***p* value^*^** |
| **NO. of patients** | **85** | **22** |  | **40** | **21** |  |
| **Median Age, years (range)** | 67 ( 40-85 ) | 62.5 ( 40-83 ) | 0.411 | **63.5 59-68.5** | **63 56-74** | **0.785** |
| **Age Group** |  |  |  |  |  |  |
| age<65 | 34 ( 40.0% ) | 13 ( 59.1% ) | 0.108 | 23 ( 57.5% ) | 12 ( 57.1% ) | 0.979 |
| age>65 | 51 ( 60.0% ) | 9 ( 40.9% ) |  | 17 ( 42.5% ) | 9 ( 42.9% ) |  |
| **Sex** |  |  |  |  |  |  |
| female | 15 ( 17.6% ) | 6 ( 27.3% ) | 0.311 | 8 ( 20.0% ) | 5 ( 23.8% ) | 0.730 |
| male | 70 ( 82.4% ) | 16 ( 72.7% ) |  | 32 ( 80.0% ) | 16 ( 76.2% ) |  |
| **BMI** |  |  |  |  |  |  |
| <25 | 66 ( 77.6% ) | 16 ( 72.7% ) | 0.627 | 34 ( 85.0% ) | 15 ( 71.4% ) | 0.205 |
| >25 | 19 ( 22.4% ) | 6 ( 27.3% ) |  | 6 ( 15.0% ) | 6 ( 28.6% ) |  |
| **ECOG** |  |  |  |  |  |  |
| 0 or 1 | 66 ( 77.6% ) | 16 ( 72.7% ) | 0.627 | 35 ( 87.5% ) | 18 ( 85.7% ) | 1.000¶ |
| >=2 | 19 ( 22.4% ) | 6 ( 27.3% ) |  | 5 ( 12.5% ) | 3 ( 14.3% ) |  |
| **Smoke** |  |  |  |  |  |  |
| never | 15 ( 17.6% ) | 8 ( 36.4% ) | 0.057 | 11 ( 27.5% ) | 8 ( 38.1% ) | 0.396 |
| ex or current | 70 ( 82.4% ) | 14 ( 63.6% ) |  | 29 ( 72.5% ) | 13 ( 61.9% ) |  |
| **CCI** |  |  |  |  |  |  |
| <10 | 43 ( 50.6% ) | 14 ( 63.6% ) | 0.274 | 20 ( 50.0% ) | 13 ( 61.9% ) | 0.375 |
| >=10 | 42 ( 49.4% ) | 8 ( 36.4% ) |  | 20 ( 50.0% ) | 8 ( 38.1% ) |  |
| **T** |  |  |  |  |  |  |
| T1 | 3 ( 3.5% ) | 0 ( 0.0% ) | 0.344¶ | 2 ( 5.0% ) | 0 ( 0.0% ) | 0.478¶ |
| T2 | 11 ( 12.9% ) | 6 ( 27.3% ) |  | 6 ( 15.0% ) | 6 ( 28.6% ) |  |
| T3 | 16 ( 18.8% ) | 4 ( 18.2% ) |  | 8 ( 20.0% ) | 4 ( 19.0% ) |  |
| T4 | 55 ( 64.7% ) | 12 ( 54.5% ) |  | 24 ( 60.0% ) | 11 ( 52.4% ) |  |
| **N** |  |  |  |  |  |  |
| N0 | 12 ( 14.1% ) | 4 ( 18.2% ) | 0.834¶ | 6 ( 15.0% ) | 3 ( 14.3% ) | 0.997¶ |
| N1 | 6 ( 7.1% ) | 2 ( 9.1% ) |  | 4 ( 10.0% ) | 2 ( 9.5% ) |  |
| N2 | 36 ( 42.4% ) | 7 ( 31.8% ) |  | 14 ( 35.0% ) | 7 ( 33.3% ) |  |
| N3 | 31 ( 36.5% ) | 9 ( 40.9% ) |  | 16 ( 40.0% ) | 9 ( 42.9% ) |  |
| **M** |  |  |  |  |  |  |
| M1a | 44 ( 51.8% ) | 8 ( 36.4% ) | 0.198 | 17 ( 42.5% ) | 8 ( 38.1% ) | 0.740 |
| M1b | 41 ( 48.2% ) | 14 ( 63.6% ) |  | 23 ( 57.5% ) | 13 ( 61.9% ) |  |
| **Brain metastasis** |  |  |  |  |  |  |
| no | 79 ( 92.9% ) | 19 ( 86.4% ) | 0.387¶ | 38 ( 95.0% ) | 19 ( 90.5% ) | 0.602¶ |
| yes | 6 ( 7.1% ) | 3 ( 13.6% ) |  | 2 ( 5.0% ) | 2 ( 9.5% ) |  |
| **Lung metastasis** |  |  |  |  |  |  |
| no | 56 ( 65.9% ) | 13 ( 59.1% ) | 0.553 | 27 ( 67.5% ) | 13 ( 61.9% ) | 0.662 |
| yes | 29 ( 34.1% ) | 9 ( 40.9% ) |  | 13 ( 32.5% ) | 8 ( 38.1% ) |  |
| **Bone metastasis** |  |  |  |  |  |  |
| no | 58 ( 68.2% ) | 13 ( 59.1% ) | 0.418 | 20 ( 50.0% ) | 12 ( 57.1% ) | 0.596 |
| yes | 27 ( 31.8% ) | 9 ( 40.9% ) |  | 20 ( 50.0% ) | 9 ( 42.9% ) |  |
| **Pleural metastasis** |  |  |  |  |  |  |
| no | 34 ( 40.0% ) | 6 ( 27.3% ) | 0.271 | 15 ( 37.5% ) | 6 ( 28.6% ) | 0.486 |
| yes | 51 ( 60.0% ) | 16 ( 72.7% ) |  | 25 ( 62.5% ) | 15 ( 71.4% ) |  |
| **Liver metastasis** |  |  |  |  |  |  |
| no | 76 ( 89.4% ) | 18 ( 81.8% ) | 0.462¶ | 35 ( 87.5% ) | 17 ( 81.0% ) | 0.706¶ |
| yes | 9 ( 10.6% ) | 4 ( 18.2% ) |  | 5 ( 12.5% ) | 4 ( 19.0% ) |  |
| **Pericardial metastasis** |  |  |  |  |  |  |
| no | 74 ( 87.1% ) | 18 ( 81.8% ) | 0.528¶ | 32 ( 80.0% ) | 17 ( 81.0% ) | 1.000¶ |
| yes | 11 ( 12.9% ) | 4 ( 18.2% ) |  | 8 ( 20.0% ) | 4 ( 19.0% ) |  |
| **Adrenal metastasis** |  |  |  |  |  |  |
| no | 76 ( 89.4% ) | 18 ( 81.8% ) | 0.462¶ | 36 ( 90.0% ) | 17 ( 81.0% ) | 0.429¶ |
| yes | 9 ( 10.6% ) | 4 ( 18.2% ) |  | 4 ( 10.0% ) | 4 ( 19.0% ) |  |
| **No of metastatic organs** |  |  |  |  |  |  |
| <2 | 46 ( 54.1% ) | 6 ( 27.3% ) | 0.025 | 12 ( 30.0% ) | 6 ( 28.6% ) | 0.907 |
| >= 2 | 39 ( 45.9% ) | 16 ( 72.7% ) |  | 28 ( 70.0% ) | 15 ( 71.4% ) |  |
| **Chemotherapy** |  |  |  |  |  |  |
| Single | 26 ( 30.6% ) | 7 ( 31.8% ) | 0.911 | 17 ( 42.5% ) | 7 ( 33.3% ) | 0.486 |
| Doublet | 59 ( 69.4% ) | 15 ( 68.2% ) |  | 23 ( 57.5% ) | 14 ( 66.7% ) |  |
| **Regimens** |  |  |  |  |  |  |
| Gem-based | 31 ( 36.5% ) | 7 ( 31.8% ) | 0.470 | 15 ( 37.5% ) | 6 ( 28.6% ) | 0.781 |
| Doc-based | 34 ( 40.0% ) | 7 ( 31.8% ) |  | 12 ( 30.0% ) | 7 ( 33.3% ) |  |
| Vin-based | 20 ( 23.5% ) | 8 ( 36.4% ) |  | 13 ( 32.5% ) | 8 ( 38.1% ) |  |
| **Surgery of primary tumor** | yes 78 ( 91.8% ) | 20 ( 90.9% ) | 1.000¶ | 36 ( 90.0% ) | 19 ( 90.5% ) | 1.000¶ |
| no | 7 ( 8.2% ) | 2 ( 9.1% ) |  | 4 ( 10.0% ) | 2 ( 9.5% ) |  |
| **Radiation therapy** |  |  |  |  |  |  |
| yes | 61 ( 71.8% ) | 13 ( 59.1% ) | 0.251 | 32 ( 80.0% ) | 12 ( 57.1% ) | 0.112 |
| no | 24 ( 28.2% ) | 9 ( 40.9% ) |  | 8 ( 20.0% ) | 9 ( 42.9% ) |  |
| ^*^ All *p* values for the categorical variables are analyzed by the Log-rank test. | | | | | | |
| ¶ Fisher’s exact test | | | | | | |
